# Supplementary material for: Technology-mediated screening interviews for youth mental health: Content validation, randomized controlled trial, and expert evaluation
Source: PLOS Digit Health. 2026 Apr 3;5(4):e0001069. doi: 10.1371/journal.pdig.0001069 (PMC13048375; doi:10.1371/journal.pdig.0001069)
Supplement: S1 Table — (DOCX) [file pdig.0001069.s001.docx]

S1 Table. Summary of the qualitative responses to the first version of the interview questions (Study 1).

| **Interview question** | **N of comments** | **Main themes in comments** |
| --- | --- | --- |
| 1. Can you tell me about yourself, so I can get an idea of who you are? | 1 | Too general, suggest more specific questions |
| 2. How would you describe yourself to someone who is just getting to know you? Include your feelings, interests, and relationships in the description as well. | 4 | Too broad, need specificity/examples, especially for emotions/relationships, better wording |
| 3. Can you share what goes through your mind on a regular day, including any persistent negative thoughts or worries? | 3 | Overly general, hard to answer, needs more concrete situations |
| 4. How would you describe your mood and energy levels in recent weeks? Have you noticed any changes affecting your daily life, work or school, family, or friends? | 5 | Vague terms/timing, too complex as is, needs specification and splitting |
| 5. Can you describe your usual response to difficult situations, especially in relation to others? | 6 | "Difficult" is vague, examples needed, recommend specificity, avoid yes/no |
| 6. When you’re not feeling well, do you engage in any activities or hobbies? Does this help you feel better? | 2 | Split into two, avoid yes/no, use open questioning |
| 7. Is there anything specific that you find really fascinating or intriguing lately? And how did that make you feel? | 2 | Avoid yes/no, specify time frame, recommend open form |
| 8. How do you cope with stress or situations that overwhelm you? Can you recall any recent achievement or area you are proud of? | 5 | Should split, topics unrelated, better as two questions |
| General comments | 3 | Keep questions simple and specific, avoid compound questions, specify context |
