# Supplementary material for: Technology-mediated screening interviews for youth mental health: Content validation, randomized controlled trial, and expert evaluation
Source: PLOS Digit Health. 2026 Apr 3;5(4):e0001069. doi: 10.1371/journal.pdig.0001069 (PMC13048375; doi:10.1371/journal.pdig.0001069)
Supplement: S2 Table — (DOCX) [file pdig.0001069.s002.docx]

S2 Table. Diagnoses of participants for the total sample and by experimental conditions (Study 2).

| **ICD-10 diagnoses** | | **Total sample** | | **By experimental condition** | | | | | |
| --- | --- | --- | --- | --- | --- | --- | --- | --- | --- |
|  |  |  |  | **Psychiatrist** | | **Chatbot** | | **Robot** | |
|  |  | f | % | f | % | f | % | f | % |
| F12 | Cannabis related disorders | 3 | 2.83 |  |  | 2 | 5.88 | 1 | 2.70 |
| F19 | Other psychoactive substance related disorers | 2 | 1.89 | 1 | 2.86 | 1 | 2.94 |  |  |
| F32 | Depressive episode | 15 | 14.15 | 2 | 5.71 | 4 | 11.76 | 9 | 24.32 |
| F40 | Phobic anxiety disorders | 1 | 0.94 |  |  |  |  | 1 | 2.70 |
| F41 | Other anxiety disorders | 20 | 18.87 | 7 | 20.00 | 8 | 23.53 | 5 | 13.51 |
| F42 | Obsessive-compulsive disorder | 6 | 5.66 | 3 | 8.57 | 1 | 2.94 | 2 | 5.41 |
| F43 | Reaction to severe stress, and adjustment disorders | 6 | 5.66 | 2 | 5.71 | 2 | 5.88 | 2 | 5.41 |
| F48 | Other nonpsychotic mental disorders | 1 | 0.94 |  |  | 1 | 2.94 |  |  |
| F50 | Eating disorders | 18 | 16.98 | 7 | 20.00 | 3 | 8.82 | 8 | 21.62 |
| F60 | Specific personality disorders | 17 | 16.04 | 6 | 17.14 | 7 | 20.59 | 4 | 10.81 |
| F81 | Specific developmental disorders of scholastic skills | 1 | 0.94 |  |  |  |  | 1 | 2.70 |
| F84 | Pervasive developmental disorders | 2 | 1.89 |  |  |  |  | 2 | 5.41 |
| F90 | Attention-deficit hyperactivity disorders | 14 | 13.21 | 3 | 8.57 | 4 | 11.76 | 7 | 18.92 |
| F91 | Conduct disorders | 1 | 0.94 |  |  |  |  | 1 | 2.70 |
| F92 | Mixed disorders of conduct and emotions | 17 | 16.04 | 7 | 20.00 | 6 | 17.65 | 4 | 10.81 |
| F93 | Emotional disorders with onset specific to childhood | 8 | 7.55 | 1 | 2.86 | 5 | 14.71 | 2 | 5.41 |
| F95 | Tic disorder | 1 | 0.94 |  |  |  |  | 1 | 2.70 |
| F98 | Other behavioral and emotional disorders with onset usually occurring in childhood and adolescence | 7 | 6.60 | 3 | 8.57 | 2 | 5.88 | 2 | 5.41 |
| R45 | Symptoms and signs involving emotional state | 14 | 13.21 | 6 | 17.14 | 4 | 11.76 | 4 | 10.81 |
| R46 | Symptoms and signs involving appearance and behavior | 5 | 4.72 | 1 | 2.86 | 3 | 8.82 | 1 | 2.70 |
| R48 | Dyslexia and other symbolic dysfunctions, not elsewhere classified | 1 | 0.94 |  |  |  |  | 1 | 2.70 |
| Z03 | Encounter for medical observation for suspected diseases and conditions ruled out | 1 | 0.94 |  |  | 1 | 2.94 |  |  |
| Z60 | Problems related to social environment | 1 | 0.94 |  |  | 1 | 2.94 |  |  |
| Z61 | Problems related to negative life events in childhood | 2 | 1.89 | 2 | 5.71 |  |  |  |  |
| Z63 | Other problems related to primary support group, including family circumstances | 3 | 2.83 | 1 | 2.86 | 2 | 5.88 |  |  |
| Z91 | Personal risk factors, not elsewhere classified | 5 | 4.72 | 2 | 5.71 | 1 | 2.94 | 2 | 5.41 |
|  | No diagnosis | 10 | 9.43 | 5 | 14.29 | 4 | 11.76 | 1 | 2.70 |

*Notes.* Percentages are calculated in reference to the whole sample size or sample sizes in each of the conditions (Total sample – N = 106; Psychiatrist – N = 35; Chatbot – N = 34; Robot – N = 37), respectively. The sum of percentages per experimental condition exceeds 100%, as several participants had more than one diagnosis (Psychiatrist: min = 1, max = 5, M = 1.8, SD = 1.0; Chatbot: min = 1, max = 5, M = 1.9, SD = 1.0; Robot: min = 1, max = 4, M = 1.7, SD = 0.9)
