# Supplementary material for: Technology-mediated screening interviews for youth mental health: Content validation, randomized controlled trial, and expert evaluation
Source: PLOS Digit Health. 2026 Apr 3;5(4):e0001069. doi: 10.1371/journal.pdig.0001069 (PMC13048375; doi:10.1371/journal.pdig.0001069)
Supplement: S3 Table — (DOCX) [file pdig.0001069.s003.docx]

S3 Table. Descriptive statistics of and correlations between the central variables – total sample (Study 2).

|  |  | **M** | **SD** | **1** | **2** | **3** | **4** | **5** | **6** | **7** | **8** | **9** | **10** | **11** | **12** |
| --- | --- | --- | --- | --- | --- | --- | --- | --- | --- | --- | --- | --- | --- | --- | --- |
| 1 | Extraversion | 2.82 | 0.95 |  |  |  |  |  |  |  |  |  |  |  |  |
| 2 | Agreeableness | 3.63 | 0.65 | .02 |  |  |  |  |  |  |  |  |  |  |  |
| 3 | Conscientiousness | 3.21 | 0.86 | .35** | .20* |  |  |  |  |  |  |  |  |  |  |
| 4 | Negative emotionality | 3.71 | 0.83 | -.34** | -.18* | -.31** |  |  |  |  |  |  |  |  |  |
| 5 | Openness | 3.53 | 0.84 | .22* | .13 | .04 | .01 |  |  |  |  |  |  |  |  |
| 6 | Self-deceptive enhancement | 3.82 | 1.01 | .42** | .17* | .42** | -.50** | .21* |  |  |  |  |  |  |  |
| 7 | Impression management | 4.55 | 1.14 | .05 | .53** | .34** | -.18* | 0.12 | .34** |  |  |  |  |  |  |
| 8 | Satisfaction with communication | 3.38 | 0.76 | .13 | .16* | .30** | -.08 | .19* | .06 | .14 |  |  |  |  |  |
| 9 | Satisfaction with the interview | 3.68 | 1.17 | .08 | .26** | .18* | -.11 | .20* | .06 | .13 | .51** |  |  |  |  |
| 10 | Willingness to repeat the interview | 1.64 | 0.69 | -.15 | -.03 | -.16 | .01 | -.10 | -.09 | .03 | -.37** | -.27** |  |  |  |
| 11 | Willingness to repeat the interview - frequency | 3.02 | 1.45 | -.03 | .07 | -.10 | .03 | -.22* | -.10 | .02 | -.50** | -.37** | .56** |  |  |
| 12 | Technology affinity | 4.14 | 1.42 | -.03 | -.14 | -.16 | .08 | .10 | -.10 | -.06 | -.06 | -0.02 | -.04 | -.07 |  |

*Notes.* ^a^ Lower score indicates higher willingness of conducting the pre-screening interview. ^b^ Lower score indicates willingness to conduct the pre-screening interview with higher frequency. ** p* < .05, ** *p* < .01, *** p < .001.
