# Supplementary material for: Technology-mediated screening interviews for youth mental health: Content validation, randomized controlled trial, and expert evaluation
Source: PLOS Digit Health. 2026 Apr 3;5(4):e0001069. doi: 10.1371/journal.pdig.0001069 (PMC13048375; doi:10.1371/journal.pdig.0001069)
Supplement: S4 Table — (DOCX) [file pdig.0001069.s004.docx]

S4 Table. Descriptive statistics of and correlations between the central variables – Psychiatrist condition (Study 2).

|  |  | **M** | **SD** | **1** | **2** | **3** | **4** | **5** | **6** | **7** | **8** | **9** | **10** | **11** | **12** |
| --- | --- | --- | --- | --- | --- | --- | --- | --- | --- | --- | --- | --- | --- | --- | --- |
| 1 | Extraversion | 2.76 | 0.85 |  |  |  |  |  |  |  |  |  |  |  |  |
| 2 | Agreeableness | 3.79 | 0.55 | .05 |  |  |  |  |  |  |  |  |  |  |  |
| 3 | Conscientiousness | 3.37 | 0.72 | .17 | .44** |  |  |  |  |  |  |  |  |  |  |
| 4 | Negative emotionality | 3.70 | 0.88 | -.39* | -.39* | -.42** |  |  |  |  |  |  |  |  |  |
| 5 | Openess | 3.45 | 0.84 | .57** | -.02 | -.05 | -.01 |  |  |  |  |  |  |  |  |
| 6 | Self-deceptive enhancement | 3.79 | 1.02 | .49** | .50** | .47** | -.69** | .23 |  |  |  |  |  |  |  |
| 7 | Impression management | 4.73 | 0.98 | .00 | .68** | .36* | -.37* | -.11 | .52** |  |  |  |  |  |  |
| 8 | Satisfaction with communication | 3.92 | 0.64 | .45** | .02 | 0.23 | -.20 | .28 | .32* | .05 |  |  |  |  |  |
| 9 | Satisfaction with the interview | 4.06 | 1.11 | .37* | .00 | 0.28 | -.22 | .20 | .17 | -.25 | .28 |  |  |  |  |
| 10 | Willingness to repeat the interview | 1.49 | 0.61 | -.21 | -.27 | -.29* | .27 | -.07 | -.34* | -.12 | -.36* | -.39* |  |  |  |
| 11 | Willingness to repeat the interview - frequency | 2.86 | 1.17 | -.16 | .07 | -.11 | -.08 | -.12 | -.05 | .17 | -.48** | -.13 | .51** |  |  |
| 12 | Technology affinity | 3.86 | 1.26 | .10 | .00 | -.02 | .11 | .11 | .12 | .20 | .11 | .17 | -.25 | -.03 |  |

*Notes.* ^a^ Lower score indicates higher willingness of conducting the pre-screening interview. ^b^ Lower score indicates willingness to conduct the pre-screening interview with higher frequency. ** p* < .05, ** *p* < .01, *** p < .001.
