# Supplementary material for: Technology-mediated screening interviews for youth mental health: Content validation, randomized controlled trial, and expert evaluation
Source: PLOS Digit Health. 2026 Apr 3;5(4):e0001069. doi: 10.1371/journal.pdig.0001069 (PMC13048375; doi:10.1371/journal.pdig.0001069)
Supplement: S5 Table — (DOCX) [file pdig.0001069.s005.docx]

S5 Table. Descriptive statistics of and correlations between the central variables – Chatbot condition (Study 2).

|  |  | **M** | **SD** | **1** | **2** | **3** | **4** | **5** | **6** | **7** | **8** | **9** | **10** | **11** | **12** |
| --- | --- | --- | --- | --- | --- | --- | --- | --- | --- | --- | --- | --- | --- | --- | --- |
| 1 | Extraversion | 2.83 | 0.93 |  |  |  |  |  |  |  |  |  |  |  |  |
| 2 | Agreeableness | 3.54 | 0.72 | -.19 |  |  |  |  |  |  |  |  |  |  |  |
| 3 | Conscientiousness | 3.30 | 0.89 | .41** | .12 |  |  |  |  |  |  |  |  |  |  |
| 4 | Negative emotionality | 3.63 | 0.89 | -.39* | -.14 | -.26 |  |  |  |  |  |  |  |  |  |
| 5 | Openess | 3.57 | 0.93 | -.08 | .33* | -.09 | .04 |  |  |  |  |  |  |  |  |
| 6 | Self-deceptive enhancement | 3.97 | 0.90 | .20 | 0.23 | .25 | -.37* | .08 |  |  |  |  |  |  |  |
| 7 | Impression management | 4.65 | 1.23 | .19 | .51** | .41** | -.11 | .40** | .25 |  |  |  |  |  |  |
| 8 | Satisfaction with communication | 3.01 | 0.75 | .01 | .26 | .27 | .10 | .23 | -.08 | .32* |  |  |  |  |  |
| 9 | Satisfaction with the interview | 3.26 | 1.26 | -.15 | .52** | .00 | -.03 | .20 | -.08 | .46** | .44** |  |  |  |  |
| 10 | Willingness to repeat the interview | 1.71 | 0.68 | .07 | .09 | -.12 | -.26 | -.24 | .13 | -.03 | -.31* | .06 |  |  |  |
| 11 | Willingness to repeat the interview - frequency | 3.09 | 1.51 | .19 | -.17 | -.01 | -.07 | -.32* | .11 | -.10 | -.57** | -.38* | .53** |  |  |
| 12 | Technology affinity | 4.35 | 1.50 | .00 | -.13 | -.23 | .10 | -.08 | -.27 | -.19 | -.08 | -.07 | .08 | .12 |  |

*Notes.* ^a^ Lower score indicates higher willingness of conducting the pre-screening interview. ^b^ Lower score indicates willingness to conduct the pre-screening interview with higher frequency. ** p* < .05, ** *p* < .01, *** p < .001.
