# Supplementary material for: Technology-mediated screening interviews for youth mental health: Content validation, randomized controlled trial, and expert evaluation
Source: PLOS Digit Health. 2026 Apr 3;5(4):e0001069. doi: 10.1371/journal.pdig.0001069 (PMC13048375; doi:10.1371/journal.pdig.0001069)
Supplement: S6 Table — (DOCX) [file pdig.0001069.s006.docx]

S6 Table. Descriptive statistics of and correlations between the central variables – Robot condition (Study 2).

|  |  | **M** | **SD** | **1** | **2** | **3** | **4** | **5** | **6** | **7** | **8** | **9** | **10** | **11** | **12** |
| --- | --- | --- | --- | --- | --- | --- | --- | --- | --- | --- | --- | --- | --- | --- | --- |
| 1 | Extraversion | 2.87 | 1.09 |  |  |  |  |  |  |  |  |  |  |  |  |
| 2 | Agreeableness | 3.57 | 0.65 | 0.19 |  |  |  |  |  |  |  |  |  |  |  |
| 3 | Conscientiousness | 2.96 | 0.91 | .44** | .11 |  |  |  |  |  |  |  |  |  |  |
| 4 | Negative emotionality | 3.80 | 0.75 | -.29* | -.05 | -0.24 |  |  |  |  |  |  |  |  |  |
| 5 | Openess | 3.57 | 0.77 | 0.23 | .05 | .28* | -.01 |  |  |  |  |  |  |  |  |
| 6 | Self-deceptive enhancement | 3.70 | 1.10 | .53** | -.10 | .50** | -.43** | .32* |  |  |  |  |  |  |  |
| 7 | Impression management | 4.27 | 1.16 | -.01 | .45** | .20 | -.05 | .03 | .26 |  |  |  |  |  |  |
| 8 | Satisfaction with communication | 3.20 | 0.60 | .13 | -.08 | .39** | -.25 | .27 | .06 | -.08 |  |  |  |  |  |
| 9 | Satisfaction with the interview | 3.70 | 1.02 | .10 | .05 | .32* | -.13 | .27 | .14 | .07 | .70** |  |  |  |  |
| 10 | Willingness to repeat the interview | 1.73 | 0.77 | -.29* | .09 | -.07 | .02 | -.03 | -.06 | .23 | -.38** | -.46** |  |  |  |
| 11 | Willingness to repeat the interview - frequency | 3.11 | 1.65 | -.13 | .32* | -.15 | .23 | -.22 | -.28* | .06 | -.56** | -.56** | .59** |  |  |
| 12 | Technology affinity | 4.22 | 1.47 | -.16 | -.19 | -.16 | .05 | .26 | -.16 | -.09 | .04 | -.01 | -.05 | -.29* |  |

*Notes.* ^a^ Lower score indicates higher willingness of conducting the pre-screening interview. ^b^ Lower score indicates willingness to conduct the pre-screening interview with higher frequency. ** p* < .05, ** *p* < .01, *** p < .001.
