# Supplementary material for: Technology-mediated screening interviews for youth mental health: Content validation, randomized controlled trial, and expert evaluation
Source: PLOS Digit Health. 2026 Apr 3;5(4):e0001069. doi: 10.1371/journal.pdig.0001069 (PMC13048375; doi:10.1371/journal.pdig.0001069)
Supplement: S7 Table — (DOCX) [file pdig.0001069.s007.docx]

Supplementary Table 7. Predictors of satisfaction with and willingness to repeat the screening interview in the samples conducting interviews with the help of technology.

|  | **Satisfaction with the interview** | **Satisfaction with communication** | **Willingness to repeat the interview** | **Willingness to repeat the interview - frequency** |
| --- | --- | --- | --- | --- |
| Gender | -0.04 | 0.04 | 0.08 | 0.18 |
| Age | -0.19 | -0.19 | 0.12 | 0.05 |
| Extraversion | -0.14 | -0.06 | -0.12 | 0.13 |
| Agreeableness | 0.23 | 0.04 | 0.06 | 0.12 |
| Conscientiousness | 0.06 | 0.32 | -0.06 | -0.07 |
| Negative emotionality | -0.05 | -0.02 | -0.15 | 0.05 |
| Open-mindedness | 0.16 | 0.24 | -0.17 | -0.28 |
| Self-deceptive enhancement | -0.09 | -0.29 | 0.07 | 0.00 |
| Impression management | 0.07 | 0.05 | 0.09 | -0.05 |
| Technology affinity | -0.02 | -0.04 | 0.05 | -0.03 |
| *R^2^* | .17 | .23 | .10 | .14 |
| *F*^a^ | 1.15 | 1.66 | .62 | .94 |

*Notes.* Standardized betas are reported. N = 68 as we only included the participants from the Chatbot and Robot conditions, and those that identified as male (0) or female (1) in these analyses. ^a^ Degrees of freedom were 10, 57.
