# Supplementary material for: Technology-mediated screening interviews for youth mental health: Content validation, randomized controlled trial, and expert evaluation
Source: PLOS Digit Health. 2026 Apr 3;5(4):e0001069. doi: 10.1371/journal.pdig.0001069 (PMC13048375; doi:10.1371/journal.pdig.0001069)
Supplement: S8 Table — (DOCX) [file pdig.0001069.s008.docx]

S8 Table. Questions for the evaluation of pre-screening interview videos by experts (Study 3).

| **Topic** | **Item** | **Variable name** |
| --- | --- | --- |
| (1) Relevance and completeness of information | 1. How comprehensive is the observed pre-screening interview in capturing the adolescent’s broader psychosocial factors? | Coverage of psychosocial factors |
| (2) Clinical usefulness and significance for treatment planning | 1. How well does the information obtained in the observed pre-screening interview contribute to formulating an accurate diagnosis? | Contribution to diagnosis accuracy |
|  | 2. Does the information obtained in the observed pre-screening interview assist in setting individualized treatment goals? | Support for setting personalized treatment goals |
|  | 3. Does the information obtained in the observed pre-screening interview help in selecting appropriate interventions? | Aid in choosing appropriate interventions |
|  | 4. How meaningful does the use of the observed pre-screening interview before each session seem for monitoring the child’s progress over time? | Suitability for ongoing progress monitoring |
|  | 5. How meaningful does the use of the observed pre-screening interview seem for identifying individuals at higher risk of developing mental disorders? | Usefulness in identifying high-risk individuals |
| (3) Integration with clinical assessment | 1. Does the observed pre-screening interview complement and enhance the overall clinical assessment process? | Complement and enhancement of overall clinical evaluation |
|  | 2. How well do the findings from the observed pre-screening interview correspond with other methods of clinical assessment? | Alignment with other assessment methods |
