# Supplementary material for: Technology-mediated screening interviews for youth mental health: Content validation, randomized controlled trial, and expert evaluation
Source: PLOS Digit Health. 2026 Apr 3;5(4):e0001069. doi: 10.1371/journal.pdig.0001069 (PMC13048375; doi:10.1371/journal.pdig.0001069)
Supplement: S9 Table — (DOCX) [file pdig.0001069.s009.docx]

S9 Table. Comparisons of mean evaluations of variables by four experts (study 3).

|  | **Psychiatrist** | | | **Chatbot** | | | **Robot** | | |
| --- | --- | --- | --- | --- | --- | --- | --- | --- | --- |
|  | **F** | **df** | **p** | **F** | **df** | **p** | **F** | **df** | **p** |
| Coverage of psychosocial factors | ^a^ |  |  | 0.742 | (3, 11.0) | 0.549 | 0.325 | (3, 8.2) | 0.807 |
| Contribution to diagnosis accuracy | 0.117 | (2, 8.4) | 0.891 | ^a^ |  |  | 1.027 | (3, 8.4) | 0.429 |
| Support for setting personalized treatment goals | 0.327 | (2, 7.8) | 0.731 | 0.463 | (3, 13.2) | 0.713 | 0.906 | (3, 8.4) | 0.478 |
| Aid in choosing appropriate interventions | ^a^ |  |  | 1.465 | (3, 11.5) | 0.275 | 0.316 | (3, 8.3) | 0.813 |
| Suitability for ongoing progress monitoring | 0.322 | (2, 8.0) | 0.734 | 0.302 | (3, 13.6) | 0.824 | 0.426 | (3, 9.0) | 0.739 |
| Usefulness in identifying high-risk individuals | 0.633 | (2, 8.0) | 0.556 | 4.57 | (3, 13.0) | 0.021 | 1.001 | (3, 8.5) | 0.438 |
| Complement and enhancement of overall clinical evaluation | 15.61 | (2, 7.5) | 0.002 | 2.175 | (3, 10.9) | 0.149 | 1.307 | (3, 8.0) | 0.338 |
| Alignment with other assessment methods | ^a^ |  |  | 3.531 | (3, 10.5) | 0.054 | 0.324 | (3, 8.0) | 0.808 |

*Notes.* Due to the violation of the assumption of the homogeneity of variances, Welch's ANOVA is reported. ^a^ Some comparisons were not performed due to at least one expert having 0 variance in their evaluations of the videos. The results were considered statistically significant if the *p* value was below .002 as adjusted by Bonferroni correction for multiple comparisons.
