# Supplementary material for: Technology-mediated screening interviews for youth mental health: Content validation, randomized controlled trial, and expert evaluation
Source: PLOS Digit Health. 2026 Apr 3;5(4):e0001069. doi: 10.1371/journal.pdig.0001069 (PMC13048375; doi:10.1371/journal.pdig.0001069)
Supplement: Attachment — Submitted filename: response-to-reviewers.docx [file pdig.0001069.s014.docx]

Dear Reviewer,

We appreciate the thoughtful and thorough review of our manuscript on technology-mediated screening interviews for youth mental health assessment. The feedback provided has been invaluable in helping us refine our work and address important considerations for this research area. We have considered all suggestions and have made substantive revisions to strengthen our study's methodology, analysis, and presentation. Where appropriate, we have expanded our discussion to better contextualize findings and acknowledge limitations.

Below is the detailed response on how we have addressed each concern raised during the review process.

**R1. Clarify “willingness” item meaning**

**Reviewer's Comment:** “It needs to be mentioned clearly that the willingness refers to willingness to repeat the interview from the same condition as the participant first participated, not willingness to repeat the interview in any of the other two modalities.”

**Authors’ Response:**

Thank you for this remark. In Study 2, the “willingness to repeat the interview” items were administered immediately after the screening interview; therefore, they refer to repeating the interview in the same modality/condition the participant had just experienced (psychiatrist, chatbot, or robot).

We have revised the wording of H4 accordingly to make this explicit:

*“Willingness to repeat the interview with the same type of conversation just completed (in the assigned condition: psychiatrist-, chatbot-, or robot-delivered) will not significantly differ between the three conditions, supporting the feasibility of technology-mediated solutions from the perspective of children and adolescents.”*

**R2. Move recruitment description to Methods**

**Reviewer's Comment**: “Move to methods.”

**Authors’ Response:**

We have moved the description of the recruitment process for Studies 1, 2 and 3 to the Methods section and created a new subsection: **2.3 Recruitment**.

**R3. Define CVI and CVR at first use**

**Reviewer's Comment**: “First mention of abbreviations CVI and CVR need to be explained here.”

**Authors’ Response:**

Thank you for noting this. We now define CVI (content validity index) and CVR (content validity ratio) in the main text at their first occurrence, rather than only in the Table 1 footnote.

**R4. Introduce interview questions**

**Reviewer's Comment**: ”Introduce interview questions in Methods before Table 1”

**Authors’ Response:**

Thank you for this suggestion. We revised Section 2.1.1 (Study 1: Content Validity Assessment) and added the following paragraph to introduce the interview questions before Table 1:

“A brief screening interview was co-developed with child and adolescent psychiatrists and psychologists to elicit contextual and observable information that can complement standardized screening tools in routine clinical practice (e.g., clarifying situational context, onset/timeline, and behavioral/interactional cues). The initial interview consisted of eight open-ended questions developed in collaboration with child and adolescent psychiatrists and psychologists to reflect a standard introductory clinical interview. The questions are presented in Table 1. Based on expert feedback, some questions were subsequently refined, resulting in the final 10-question version used in Study 2. These are presented Table 2.”

**R5. Clarify “1–5 conditions” meaning**

**Reviewer's Comment**: “What does this mean (because below it’s stated more than 5 conditions)? Does this mean altogether there were 1–5 conditions the participants were diagnosed with, or that one participant in general might be diagnosed with 1–5 conditions out of a set of conditions?”

**Authors’ Response:**

This refers to the number of diagnoses per participant (i.e., comorbidity count), not the number of diagnostic categories in the overall sample. Specifically, each participant had between 0 and 5 recorded ICD-10 diagnoses (min–max per person), and 10 participants had no prior diagnosis recorded (i.e., first presentation at the clinic). We revised the text as follows:

Original: “Participants were diagnosed with from 1 to 5 conditions (M = 1.79, SD = .97), while 10 (9.4%) participants were not diagnosed with any condition…”

Revised: “Across participants, the number of diagnoses per person ranged from 0 to 5 (M = 1.79, SD = 0.97), with 10 participants (9.4%) having no prior diagnosis recorded. The most prevalent diagnoses observed in the study sample were within the following ICD-10 categories: …”

**R6. Between-subject design and power**

**Reviewer's Comment**: “As this study design was a between-subject design, lower statistical power could be due to differences between groups reflecting individual variability rather than interview type.”

**Authors’ Response:**

Thank you. Indeed this is an important fact. We addressed this in the Discussion (rather than Results) by explicitly acknowledging that Study 2 used a between-subject (independent-measures) design; thus, between-group individual variability (even with random allocation) may increase error variance, reduce statistical power, and contribute to observed differences (or lack of differences) between modalities. We added the following text:

“Since Study 2 used a between-subject design, individual-difference variation between randomized groups could have increased error variance and reduced statistical power. This may also partially account for some observed differences in satisfaction. Thus, modality effects on satisfaction with the interview should be interpreted accordingly.”

**R7. Remove repeated information (ANOVA sentence)**

**Reviewer's Comment**: “Repeated info. Format.”

**Authors’ Response:**

Thank you for the observation. We removed the repetition and replaced the two sentences with:

“Next, we conducted a one-way ANOVA comparing experts’ evaluations of the screening-interview videos across three interviewer modalities: Psychiatrist, Chatbot, and Robot.”

**R8. Replace “AI agent” with “technology-mediated”**

**Reviewer's Comment**: “Technology-assisted or technology-mediated might be more suitable, as rule-based programs are not really AI agents.”

**Authors’ Response:**

Thank you for this suggestion. We agree that describing the interviewing system as an “AI agent” could be misleading because the dialogue logic was primarily rule-based. We therefore revised the manuscript to use “technology-mediated” (and/or “technology-assisted”) terminology throughout and clarified the rule-based nature of the interviewing workflow. At the same time, however, we would like to note that the robot platform employs AI-based modules for speech/gesture synthesis and perception.

**R9. Within-subject design suggestion (washout/carryover)**

**Reviewer's Comment**: “A within-subject design for this study could have revealed the same.” (placed near the satisfaction/disclosure paragraph)

**Authors’ Response:**

Thank you for this suggestion. It is true, a within-subject (cross-over) design could increase power by controlling for between-person variability. However, implementing a within-subject design here would likely require counterbalancing and sufficiently long washout periods to mitigate carryover and order effects (e.g., familiarity with the questions, reduced novelty, and anchoring of satisfaction/disclosure ratings), which would increase participant burden and study duration. Washout periods can reduce carryover, but residual carryover may still remain a concern. Therefore, we used a between-subject design for Study 2 and added the following paragraph to the Discussion:

“Moreover, a within-subject cross-over design might be more sensitive to subtle modality effects but would require counterbalancing and washout periods to reduce carryover effects. If applied in future studies, possible residual carryover should be considered as it may affect interpretation.”

**R10. Methods should precede Results**

**Reviewer's Comment**: “Methods need to precede Results.”

**Authors’ Response:**

We reorganized the manuscript so that the Methods section appears immediately after the Introduction.

**R11. Interview questions: in Supplement but must be referenced**

**Reviewer's Comment**: “Provided in supplementary but need to be referred here” + “What were the interview questions?”

**Authors’ Response:**

Thank you for this comment. We added an explicit in-text reference in the Methods:

“Study 1 applied a structured interview. The full set of questions asked to the participants is provided in Supplementary Information (Supplementary Table 1).”

**R12. Why not have each participant do all three assessments?**

**Reviewer's Comment**: “What was the reason for not getting a participant to do all 3 kinds of assessments?”

**Authors’ Response:**

Thank you for this comment. Having each participant complete all three modalities would constitute a within-subject/cross-over design and would likely introduce order and carryover effects (e.g., learning/familiarity with the questions and modality comparisons). An appropriate cross-over design would also require counterbalancing and washout periods to minimize carryover, increasing participant burden and study duration. We clarified this rationale in the Discussion (please also see response to R9).

**R13. Why hide existence of other modalities?**

**Reviewer's Comment**: “I’m not fully convinced why it had to be hidden from the participants that there are other assessment methods. Suggest to add a clearer explanation of why this was hidden from them.”

**Authors’ Response:**

Thank you for raising this point. The study information did not emphasize the existence of multiple modalities because awareness of alternatives could introduce expectancy/demand effects (participants comparing modalities and adjusting satisfaction/disclosure/engagement accordingly), thereby confounding the modality effect. Additionally, knowing other modalities were available could increase disappointment or curiosity about “missing” conditions, influencing responses independent of the experienced modality.

We revised the blinding paragraph and added:“They were informed only about the modality they would experience. This approach was introduced to minimize cross-modality comparison effects arising from demand characteristics, disappointment, or curiosity about ‘missing’ conditions.”

**R14. Platform provenance and versions**

**Reviewer's Comments**: “Was this developed in-house… or open-source… version used?”, “robot version/model details”, “references for PLATTOS and SPREAD”.

**Authors’ Response:**

Thank you for this question. While the chatbot component is based on the open-source Rasa framework (v3.6.21), the overall platform/integration used in this study was developed in-house (not publicly released at the time of the study) refining the platform described in Mlakar et al. (2025) (https://doi.org/10.1038/s41746-025-02117-9). We revised the Methods to include implementation details, including the references and version of the robot (hardware version, Pepper v1.8 with NAOqi 2.5 SDK)

**R15. Standardised protocols vs your interview questions**

**Reviewer's Comments**: “Are there any standardised protocols/questionnaires… If so, why were they not used… and how are these questions different?”

**Authors’ Response:**

Thank you for the question. Standardized instruments for youth mental health screening and assessment exist (e.g., brief symptom questionnaires such as PHQ-9/GAD-7 and structured/semi-structured diagnostic interviews such as MINI-KID/K-SADS). However, the goal of this study was to evaluate a brief screening interview (delivered via different modalities), intended to complement (not replace) standardized tools by eliciting additional contextual and observable information that can support clinical interpretation and triage (e.g., presenting concerns in the youth’s own words, situational context, and clarification of responses). Moreover, self-reported measures can be influenced by recall/response biases and missingness/nonresponse; thus, a conversational screening with open-ended questions may provide complementary context for clinical practice. We revised the Discussion accordingly.

**R16. Referencing style inconsistency (Soto & John)**

**Reviewer's Comments**: “Inconsistent referencing styles—Soto & John reference.”

**Authors’ Response:**

Thank you for noting this. We revised the phrasing and formatting to ensure consistent citation style:

“The validation study presented in Soto & John (2017) [57] showed acceptable internal consistency…”

**R17. Cronbach’s α placement (keep in Measures)**

**Reviewer's Comments**: “Would this be more suitable in Results section?”

**Authors’ Response:**

Thank you for this suggestion. We agree that internal consistency estimates are derived from our dataset; however, we intentionally report Cronbach’s α in the Measures section to document the performance/appropriateness of the scale in our specific context and to guide interpretation of subsequent analyses (particularly given the low α observed in the Robot condition). We therefore keep this information in Measures, but revised the wording to make clear it is provided as measurement-quality context rather than a primary study outcome.
