# Supplementary material for: Technology-mediated screening interviews for youth mental health: Content validation, randomized controlled trial, and expert evaluation
Source: PLOS Digit Health. 2026 Apr 3;5(4):e0001069. doi: 10.1371/journal.pdig.0001069 (PMC13048375; doi:10.1371/journal.pdig.0001069)
Supplement: Attachment — Submitted filename: response-to-reviewers-r2.docx [file pdig.0001069.s015.docx]

Dear Editor and Reviewers,

We thank the reviewers for their constructive feedback and careful reading of our manuscript in the 2^nd^ round of the reviews. We have tried to address the comments and made corresponding revisions to further improve the work. Below, we provide a detailed point-by-point response to each reviewer's comments. All changes made to the manuscript are highlighted in the revised version for ease of review.

**Reviewer #1**

**Reviewer's Comment:** “I have one minor suggestion to improve readability. Throughout the text, the term "child and adolescent psychiatrists" is used to refer to "pediatric psychiatrists". I would use the "pediatric" to improve readability. This is nothing major and it's your choice whether to make the changes.”

**Authors’ Response:**

We thank Reviewer #1 for this suggestion. We have revised the manuscript throughout and replaced "child and adolescent psychiatrists" with "pediatric psychiatrists" to improve readability and consistency.

**Reviewer #3**

Before addressing the specific comments, we would like to apologize to Reviewer 3. Due to a technical issue with the journal’s interface, on our side, in the first review round, we inadvertently did not see and therefore did not respond to their comments. We sincerely regret this oversight and have made every effort to rectify it by addressing the remarks in this revision

**Reviewer's Comment**: “The major issue with the paper in its current form is that readers cannot meaningfully interpret the results without reading the Methods which come after the Results and Discussion. It appears that this is the journal's desired format, so it is not clear what the solution is, but readers need the information provided in the Methods section before they read the Results section, particularly with respect to the rating scales and other metrics used throughout the study and the description of the three assessment conditions.”

**Authors’ Response:**

We acknowledge this concern. The manuscript structure follows the journal's required format.

**Reviewer's Comment**: “In the initial sections of the paper the term "validation" is used too broadly. In simple terms, "validity" refers to whether the intended construct is actually measured. Only Study 1 is a validation study. The other two studies measure aspects of acceptability, feasibility, and clinical utility. It would help to clearly define these terms and use them consistently. For example, "satisfaction" is often mentioned, but it is not clear whether it is the same as acceptability or whether it has another meaning”

**Authors’ Response:**

We thank the reviewer for this remark. We have revised the Abstract and Author's Summary to reflect that only content validity was assessed in Study 1. We have also revised the Introduction section to clearly define content validity and distinguish it from acceptability, feasibility, and clinical utility. Throughout the manuscript, we have tried to use these terms consistently and clarify that Studies 2 and 3 examine acceptability, feasibility, and clinical utility rather than validation per se. We have also clarified that "satisfaction" is used as one indicator of acceptability.

**Reviewer's Comment**: ” In the Abstract and the Summary, it is not clear that there are three conditions; it seems that the tablet and robot conditions are somehow combined.”

**Authors’ Response:**

We have revised the Abstract and Author's Summary to clearly indicate that there are three distinct assessment conditions: psychiatrist-led (control), chatbot via tablet, and humanoid robot. The revised text now explicitly describes all three modalities to avoid confusion.

**Reviewer's Comment**: “Change the main Study 1, 2 and 3 titles to be more precise in Methods heading.”

**Authors’ Response:**

We have revised the Methods section headings for Studies 1, 2, and 3 to be more precise and descriptive:

•	Study 1: Content validity of the screening interview

•	Study 2: Feasibility and Acceptability from Youths' Perspective

•	Study 3: Clinical Utility from Expert Psychiatrists' Perspective

**Reviewer's Comment**: “Not all readers will be familiar with the term "2nd Bologna cycle" (page 9).”

**Authors’ Response:**

We have rephrased this to: "second-cycle (Master's-level) degree according to the Bologna system or an equivalent degree" for clarity.”

**Reviewer's Comment**: “Some of the potential problems associated with recruiting youth who are in mental health treatment are mentioned as limitations, but it seems that additional potential limitations could be associated with treatment successfully reducing or eliminating symptoms which would limit what could be detected in an assessment.”

**Authors’ Response:**

We thank the reviewer for this insightful point. We have added the following text to the Discussion:

*"An additional implication of recruiting youth already engaged in treatment is that ongoing interventions may have reduced or stabilized symptom levels by the time of participation. As a result, some clinically relevant difficulties may have been less evident during the screening interview, potentially limiting the range of symptoms that could be detected and leading to conservative estimates of the interview's ability to capture active psychopathology. Future studies should therefore also examine the performance of the screening interview in youth who are not yet in treatment or who present with more acute symptomatology."*

**Reviewer's Comment**: “It is not clear how participants responded in the chatbot and robot conditions—did they respond verbally or did they need to type their responses?”

**Authors’ Response:**

We have added clarification to Study 2 in the Methods section:

*"Participants read the chatbot's questions via the tablet and responded verbally. Their spoken answers were recorded but did not require any typing on the device."*

*"During the robot-facilitated interview, participants listened to the robot's questions and responded verbally to all questions. No typing or interaction with additional devices was required."*

**Reviewer's Comment**: “Was there any assessment of interrater reliability in Study 3? If so, it should be briefly described. If not, it should be noted as a limitation.”

**Authors’ Response:**

We thank the reviewer for raising this important point. In Study 3, we did not calculate interrater reliability because each video recording was evaluated by a single expert only; thus, there were no overlapping ratings on the same video that would allow for estimation of interrater agreement. To assess potential systematic differences between experts, we compared mean evaluations across raters and found no significant differences, as already reported in the Methods and Results of Study 3.

We have now clarified in the Methods section that each video was rated by one expert only, and we explicitly acknowledge the absence of interrater reliability estimates as a limitation in the Discussion:

*"However, we must note that in Study 3, each video was rated by a single expert only, which precluded estimation of interrater reliability. Future studies should ensure that multiple experts rate each interview video so that interrater reliability can be estimated and the robustness of expert-based clinical utility evaluations can be strengthened."*

**Reviewer's Comment**: “Several times, it is noted that a potential advantage of technology-based assessment is greater disclosure, especially among youth. However, disclosure is not formally assessed in the study. Is there anything that can be derived from the data on this topic?”

**Authors’ Response:**

We appreciate the comment. We agree that, although we discuss prior evidence suggesting that technology-mediated assessments may facilitate greater disclosure, our own study did not include a direct measure of disclosure (e.g., number or depth of sensitive disclosures, content coding of interviews). We therefore cannot empirically test the hypothesis of increased disclosure in technology conditions using the present data. We have revised the Discussion to clarify that our statements about disclosure are based on previous literature rather than on direct evidence from this study.

We have added the following text:

*"While we did not include a dedicated self-disclosure scale, communication satisfaction and willingness-to-repeat scores in the chatbot and robot conditions indicate that many adolescents felt comfortable speaking and would be willing to engage in such interviews again. This pattern offers preliminary, indirect support for the notion that technology-based pre-screening can support adolescents in sharing sensitive information, while underlining the need for future studies that incorporate dedicated, validated self-disclosure measures. This pattern is broadly in line with previous work indicating that technology-mediated or electronic assessments can provide a comfortable context for adolescents to share sensitive information."*

**R11. Interview questions: in Supplement but must be referenced**

**Reviewer's Comment**: “The accuracy of the screening is not formally discussed. Since the youth participating in the study were in treatment, it seems that results could be compared to the diagnoses obtained in treatment. Additionally, it would be useful to know if there are differences on any of the study variables associated with diagnosis, or at least, diagnostic categories.”

**Authors’ Response:**

We thank the reviewer for this important comment. The present study did not assess diagnostic accuracy of the screening interview, because no diagnoses were generated on the basis of the screening itself; accordingly, there is no interview‑derived diagnostic classification that could be directly compared with participants’ treatment diagnoses. Although we did collect existing ICD‑10 diagnoses for youth in Study 2, the clinical sample was highly heterogeneous and characterised by substantial comorbidity: across participants, the number of diagnoses per person ranged from 0 to 5 (M = 1.79, SD = 0.97), with 10 participants (9.4%) having no prior diagnosis recorded. The most prevalent diagnoses were F41 “Other anxiety disorders” (n = 20, 18.9%), F50 “Eating disorders” (n = 18, 17.0%), F60 “Specific personality disorders” (n = 17, 16.0%), F92 “Mixed disorders of conduct and emotions” (n = 17, 16.0%), F32 “Depressive episode” (n = 15, 14.2%), F90 “Attention‑deficit hyperactivity disorders” (n = 14, 13.2%), and R45 “Symptoms and signs involving emotional state” (n = 14, 13.2%), while other diagnoses appeared fewer than 10 times in the total sample. Given this pattern of overlapping diagnostic profiles and small, uneven cell sizes across categories, any post‑hoc comparisons of study variables by diagnosis or diagnostic group would be difficult to interpret and at high risk of being underpowered and arbitrary in terms of grouping strategy. We therefore did not pursue such analyses in the current manuscript. Instead, we now clarify in the Discussion that diagnostic accuracy was not examined and explicitly note the lack of diagnosis‑based analyses as a limitation, emphasising that larger future studies with more clearly defined diagnostic strata will be needed to evaluate how the screening interview performs across diagnostic categories and in relation to gold‑standard diagnostic assessments.

**Reviewer's Comment**: “It is not clear whether the chatbot or robot assessments produce any kind of report for clinicians to review and make treatment decisions. Clinical utility seems limited if it is necessary to watch a video recording of the interaction. It may be that this is a goal for future work.”

**Authors’ Response:**

We appreciate this comment. In the present study, the chatbot- and robot-mediated interviews did not generate an automated summary or structured report for clinicians; instead, expert psychiatrists in Study 3 evaluated video recordings of the interviews to rate perceived clinical utility. We agree that relying on full-length video review limits clinical utility and scalability, noting that our current findings primarily demonstrate the potential of technology-mediated interviews rather than a fully implemented clinical workflow.

Importantly, the development of automated outputs from these interviews is a key focus of our ongoing work. We are currently conducting follow-up studies in which conversational data from the screening interviews are systematically recorded and processed to extract contextual markers and generate structured, clinician-facing summaries that support contextualized, explainable decision-making. These studies will enable us to evaluate how automatically generated reports can integrate with routine care and further extend the clinical utility of technology-mediated screening.

We have revised the Future Work section to include:

*"In the current study, technology-mediated interviews did not yet produce automated reports; experts evaluated full video recordings, which constrains real-world clinical utility. Future work within our group is already underway to derive structured, explainable summaries from recorded interview data and to test how such outputs can be used by clinicians to inform assessment and treatment decisions."*

**Reviewer #4**

**Reviewer's Comment**: “All major comments have been addressed well and appropriately, and changes made throughout the manuscript. Minor formatting issues (noted in the previous review) still remain, which might be worth addressing for a good quality publication.”

**Authors’ Response:**

We appreciate the reviewer’s effort in the second iteration of the manuscript. We have now further proofread the manuscript and tried to remove or merge repeated information across paragraphs to improve clarity and readability.
